# A review of studies on community based early warning systems

**DOI:** 10.4102/jamba.v8i1.206

**Published:** 2016-04-19

**Authors:** Margaret Macherera, Moses J. Chimbari

**Affiliations:** 1 Department of Environmental Science and Health, National University of Science and Technology, Zimbabwe; 2 Department of Public health Medicine, University of KwaZulu-Natal, South Africa

## Abstract

Community-based early warning systems involve community driven collection and analysis of information that enable warning messages to help a community to react to a hazard and reduce the resulting loss or harm. Most early warning systems are designed at the national or global level. Local communities’ capacity to predict weather conditions using indigenous knowledge has been demonstrated in studies focusing on climate change and agriculture in some African countries. This review was motivated by successes made in non-disease specific community-based early warning systems with a view to identify opportunities for developing similar systems for malaria. This article reviewed the existing community-based early warning systems documented in literature. The types of disasters that are addressed by these systems and the methodologies utilised in the development of the systems were identified. The review showed that most of the documented community-based early warning systems focus on natural disasters such as floods, drought, and landslides. Community-based early warning systems for human diseases are very few, even though such systems exist at national and regional and global levels. There is a clear gap in terms of community-based malaria early warning systems. The methodologies for the development of the community-based early warning systems reviewed mainly derive from the four elements of early warning systems; namely risk knowledge, monitoring, warning communication and response capability. The review indicated the need for the development of community based early warning systems for human diseases.

## Introduction

Early warning systems are an essential component of the Community-Based Disaster Risk Management (Centre for International Studies and Cooperation 2011). They provide communities with relevant, topical information on environmental conditions so that they can assess levels of risk and make informed decisions to protect themselves. Most of the community-based early warning systems (CBEWS) are monitored by the villagers, thus empowering communities and ensuring the sustainability of the systems. Nyong, *et al*. (2007) indicate that communities affected by climate variability have developed and implemented extensive mitigation and adaptation strategies that have enabled them to reduce their vulnerability to past climate variability and change. However, this knowledge is rarely considered when designing early warning systems, particularly for the case of malaria. Very successful community-based early warning systems have been developed in the Asian countries, probably because natural disasters frequently affect these nations. Most of the community-based flood early warning systems developed in Asia are based on the four elements of people-centred early warning systems recommended by the United Nations International Strategy for Disaster Reduction (UNISDR) Platform for the Promotion of Early Warning. The four elements are knowledge of the risk; monitoring and warning services; warning dissemination and communication; and emergency response. Community-based flood early warning systems have been documented and implemented in Asia (Gautum & Phaiju 2013; Lassa & Sagala 2013; Perez, Espinueva & Hernando 2007). This review was motivated by successes made in non-disease specific community-based early warning systems and with a view to identifying opportunities for the development of similar systems for malaria. Malaria epidemics are some of the most serious public health emergencies that confront health officials, affecting highly vulnerable populations with only limited immunity to malaria (World Health Organization 2004a). Epidemic malaria is a serious problem in semi-arid and highland areas in Africa. It is estimated that epidemic malaria causes between 12% and 25% of estimated annual worldwide deaths due to malaria (Thomson *et al*. 2005). Early detection, containment and prevention of malaria constitutes one of the four technical elements of the global malaria control strategy. If epidemics are not immediately detected and controlled, they result in unacceptably high mortality and morbidity rates. A malaria epidemic may have disastrous consequences at the individual, household, community, regional and national levels. In the affected population, malaria can cause morbidity, mortality susceptibility to diseases other than malaria, and can seriously disrupt health care services. During the First southern African regional epidemic Outlook held in Harare, Zimbabwe, in 2004, member countries committed themselves to the development of integrated early warning systems as a decision support tool for improving epidemic preparedness and response planning (DaSilva *et al*. 2004). The Malaria Early Warning Systems (MEWS) Framework suggested by the World Health Organization (WHO) consists of four components (1) vulnerability monitoring, (2) seasonal climate forecasting, (3) environmental monitoring and (4) sentinel case surveillance (WHO 2004a). All these components are implemented by the health sector and its partners. Community participation is given little attention in the implementation of these activities. According to the WHO, the full practical potential of MEWS has yet to be realised. Over the past few years, attempts have been made in Africa to transform MEWS from a research project into a practical public health tool (WHO 2004b). These research projects have focused on the development of systems at regional level or country level.

Zimbabwe, like other countries of the Southern African Development Community (SADC), is committed to the Abuja targets for Roll Back Malaria in Africa, which includes improved response to epidemics. To meet the targets, countries are expected to detect 60% of malaria epidemics within 2 weeks of onset and respond to 60% of the epidemics within 2 weeks of their detection (DaSilver *et al*. 2004). This cannot be achieved without enhanced community participation and the development of early warning systems that point to where and when epidemics may occur. Community participation can be enhanced through the incorporation of their local knowledge into the malaria control programmes. Several studies have been done showing the importance of climatic factors in the development of MEWS (Ceccato *et al*. 2007; Teklehaimanot *et al*. 2004; Thomson & Connor 2001; Thomson *et al*. 2005). The studies focused on MEWS that are based on the health system with minimal involvement of the communities. Considering that local communities have the capacity to predict weather conditions using indigenous knowledge, as has been demonstrated in studies on climate change and agriculture (Kalanda-Joshua *et al*. 2011; Mudzengi *et al*. 2013; Nethononda, Odhiambo & Paterson 2013; Shoko & Shoko 2013), there is the potential for the development of community-based MEWS using indigenous knowledge systems and the general integration of indigenous knowledge systems in the existing malaria control programmes.

There has been a global commitment to the fight against malaria, as evidenced by initiatives like the Roll back Malaria, the Global Fund and the Gates Foundation. All these initiatives are foreign to most people who are affected by malaria. Some of them do not take into consideration the knowledge of local people about the disease, including ways to prevent, treat and even predict outbreaks. Diseases like malaria have existed without being considered natural disasters comparable to floods and earthquakes. Hence the capacity of the local community to predict the disease has been undermined. The top-down approach of malaria control programmes may also have contributed to the lack of community participation in the development of MEWS. Predicting malaria has been considered too complex to include the simple methods used by communities to predict diseases.

This review is based on the analysis of the existing literature on community-based early warning systems. It scrutinises the various definitions of community-based early warning systems given by various organisations and authors, and the methodologies for the development of such systems. The analysis identifies the disasters for which community-based early warning systems have been developed. The potential for developing early warning systems is explored. Early warning systems for conflict and financial crises were not considered.

## Methodology

This review is based on a systematic search of relevant literature on Google scholar (http://www.scholar.google.com) electronic search engine. The search considered scholarly literature on community-based early warning systems that have been documented globally based on the following combination of terms: ‘community based early warning systems’ OR ‘people-centred early warning systems’ OR ‘early warning systems’. The snowballing technique was used to obtain more literature based on the bibliography or reference list of papers obtained using the search strategy described above. The literature obtained included 4 books, 21 presentations from organisations dealing with early warning systems and 14 journal articles. The literature on early warning without a community-based aspect was included to strengthen the discussion and understanding of the concept and the importance of early warning systems. The literature was divided according to discussions of the following: early warning systems in general and community-based early warning systems. The community-based early warning systems were further divided into those dealing with natural disasters and those dealing with communicable diseases. The idea was to identify the types of disasters that were addressed by community-based early warning systems. Thus, definitions used by different stakeholders (researchers, policy makers, nongovernmental organisations) were analysed. The literature dealing with community-based early warning systems was further analysed for the methodologies used in the design of the systems. This helped to identify methods that could be adopted and adapted for the development of community-based early warning systems for communicable diseases.

## Results

Of the literature reviewed, 24 articles were on community-based early warning systems and the remaining 29 articles were on early warning systems either at regional or global level. Ten articles out of the 29 were on malaria early warning, while one was on animal diseases. Three articles among those that discussed community-based early warning systems discussed systems for human diseases. Only one out of the 3 articles explained the development of a community-based early warning system for a specific communicable disease.

### Definitions for early warning systems

Various definitions for early warning systems have been used by different authorities. According to the United Nations International Strategy for Disaster Reduction (2009), an early warning system is the set of capacities needed to generate and disseminate timely and meaningful warning information to enable individuals, communities and organisations threatened by a hazard to prepare and act appropriately and in sufficient time to reduce the possibility of harm or loss. In this definition, the early warning system is directed to the people who are threatened by the hazard rather than the agencies working with the affected communities. The definition also emphasises the use of existing structures, which may be structures within the communities. These structures can be used for the dissemination of information about a disaster or the mitigation measures. The source of the warning is not specified. We are of the opinion that the affected people should be able to identify the indicators for imminent dangers so that they can prepare for mitigation. This does not, however, suggest that they should have the capacity to deal with the hazard. The warning should allow them time to mobilise resources and assistance from outside, if need be. In the definitions the word ‘early’ is relative depending on the type of disaster, the community affected and the available resources to deal with the disaster. According to the Federation of Red Cross and Red Crescent Societies, (IFRC), (2013), ‘early’ signifies prior to the arrival of a hazard or threat, while there is still time to reduce the potential harm or loss or prevent a disaster. The concept of how early depends on the degree of preparedness of the people affected by a disaster and the existence of the capacity to react to the disaster. It also depends on the type of disaster, which determines the amount of time required to take protective action. The IFRC defines a warning as the message that announces the imminent danger and a system as an ordered and standardised compilation of elements that remain in constant fluctuation with movement in multiple directions. In the context of community-based early warning systems, a community represents a network of social interaction that may be exposed to multiple social and or physical impacts from one or more hazards or threats often related by place (IFRC 2013). Neither the United Nations International Strategy for Disaster Reduction, nor the IFRC definition of a community-based early warning system includes a requirement or mention of who initiates the warning under such systems. Community-based may mean that the system is at the community level but implemented by outsiders. The extent of involvement or participation of the affected communities is also absent in the definitions. Thus, the term ‘community-based’ has a wide range of meanings. According to McLeroy *et al*. (2003), community-based projects may refer to community as the setting for interventions, the target for interventions, or the resource or agent of interventions. Community-based early warning systems can be referred to as people managed, local or community centred, as defined by the UNISDR (2009). We define a community-based early warning system as one in which the communities participate in hazard identification and the formulation of the warning system, and not merely reacting to a warning at local level. Glantz (2009) argues that an early warning system is made up of many components and is not only the formulation or issuance of a warning. A holistic early warning system includes the formulation of the warning, the issuance of the warning, the reception and response to the warning and feedback to those who developed and issued the warning. According to the United Nations International Strategy for Disaster Reduction, (http://www.unisdr.org/ppew), effective early warning systems do not only need strong technical foundations and good knowledge of the risks, but should also be people centred. The systems should have clear message dissemination systems that reach those at risk and responses by risk managers and the public. The world conference on disaster reduction held in Kobe Japan in 2005 described effective early warning systems as those packaged to be understandable and relevant to the communities served (UNISDR 2005). The conference outlined the characteristics of people-centred early warning systems as the integration of a bottom-up and top-bottom element, the involvement of local communities in the early warning process, a multi-hazard approach and the cultivation of awareness in the social structure of the communities. This is a more appropriate explanation of a community-based early warning system in which the communities participate in the hazard identification and the formulation of the warning system. The people affected by the hazard are not mere recipients of warnings but are also initiators of the warnings and able to ask for any assistance, if need be. This conference was followed by the Third United Nations World Conference on Disaster Risk Reduction in Sendai Japan in March 2015 (UNISDR 2015). The conference identified a shift from disaster management to disaster risk management. The conference emphasised a more people-centred preventive approach to disaster risk. It also acknowledged that disaster risk reduction (DRR) practices need to be multi-pronged in order to address a wide range of hazards and sectors. The engagement of relevant stakeholders, including women, children and youth, persons with disabilities, indigenous groups and immigrants was also emphasised. We are of the opinion that local communities should initiate the warning for some disasters, as has been demonstrated in agriculture, because they are closer to the location of the hazard as it occurs and are the most affected. Unfortunately, local communities may be intimidated by outsiders and may not express or share their knowledge unless empowered to do so. Disempowered communities consider early warning systems an imposition, which renders the systems ineffective. Nevertheless, it should be noted that communities do not have the capacity to develop or implement early warning systems for all types of disasters. Some disasters, such as tsunamis that originate distantly, are complex and cannot be covered by a community-based system. In this article, the terms ‘people-centred’ and ‘community-based’ are used synonymously.

A complete and effective people-centred early warning system comprises of four interrelated elements: risk knowledge, monitoring and warning service, dissemination and communication and response capability (UNISDR 2009). A weakness or failure in any element could result in the failure of the whole system. This means that hazards and vulnerabilities must be well known, and their patterns and trends must be established. This enables the development of hazard monitoring and early warning services. The warnings should reach all the people at risk. The warning information should be clear and usable and the risks should be well understood. It is essential that communities understand their risks, respect the warning service and know how to react. People-centred early warning systems rely on the participation of those most likely to be at risk. A local bottom-up approach to early warning enables the communities to participate in reducing their vulnerability and strengthening their capacities. The UNISDR (2009) definition for a people-centred early warning system puts the affected people at the centre and acknowledges that they have a role in reducing their vulnerability to hazards, while also taking into account that affected populations may need outside assistance. The inclusion of a top-down element in the definition makes this point.

All the definitions identified in this review emphasise the importance of community participation in the formulation of systems and the responses triggered by them. However, the extent of the participation is not clear. Some definitions interpret community involvement as participation. Sustainability is another important aspect embedded in the definitions. Community participation creates ownership of the early warning systems and therefore enhances their sustainability. In addition, the acceptance of community-based early warning systems, just as in all early warning systems, depends on their reliability. The system should not produce false alarms or miss hazards. Most of the authors of the literature that was reviewed derived the definition of early warning systems from the UNISDR (2009) definition.

### Types of early warning systems

#### Global and national early warning systems

Early warning systems exist at different levels such as the global, national and local levels. The levels are determined by the magnitude of the problems and the capacities of the various levels to address them. Successful early warning systems have been developed for the agricultural sector and include the USAID’s Famine Early Warning System (FEWS), SADC Food Security Programme (/REWU), FAO Global Information and Early Warning System (GIEWS) on Food and Agriculture, FAO Food Insecurity and Vulnerability Information and Mapping Systems (FIVIMS) and World Food Programme (WFP) Vulnerability Analysis and Mapping (VAM). All these systems are developed at global level and communicated down to the regions and countries concerned until the warnings reach the affected populations. Nevertheless, the ordinary farmer in a village may not get some warnings unless he listens to the radio or watches television. These early warning systems are necessary for communities to understand regardless of their technological complexity. Thus, to be useful and relevant, early warning systems must be designed to suit the populations and local settings.

The WHO has promoted MEWS as a core component of epidemic risk management for many years (Cox & Abeku 2007). The importance of the use of climatic factors in the development of MEWS in Africa has been demonstrated by several authors (DaSilva *et al*. 2004; Mabaso *et al*. 2006; Teklehaimanot *et al*. 2004; Thomson *et al*. 2005).

The MEWS are targeted at alerting health systems. Even though communities are capable of predicting climatic events in their own areas, the existing MEWS do not take this knowledge into account. In response to the need to develop MEWS in Africa, the WHO published a framework proposing the generic concepts and potential early warning and detection indicators for use in MEWS (Thomson & Connor 2001). Thompson and Connor stated that ideally seasonal climate forecasts will accurately predict the average season’s weather, increases in mosquito density and survival, including mosquito and parasite development rates. Vector and parasite dynamics will accurately predict increases in the entomological inoculation rate, which will be directly related to the number of malaria cases. Thomson *et al*. (2005) indicate that rainfall anomalies are widely regarded to be a major driver of the inter-annual variability of malaria incidence in semi-arid areas of Africa. This suggests that rainfall, among other weather phenomena, can be used in the development of MEWS. Thomson and Connor (2001) suggest that seasonal climate forecasts can be used to predict an increase or decrease in the number of malaria cases, and also that rainfall is a major driver for malaria incidence. Based on these ideas, it is possible to develop a MEWS using indigenous knowledge systems as means for weather forecasting, since this knowledge has been used in early warning systems in agriculture and other natural disasters. This framework is targeted at informing the health sector on how they can develop MEWS. The MEWS advocated for by the WHO is an example of a national early warning system.

Early warning systems are continually being created as new threats are being identified (Glantz 2009). The early warning systems are, however, becoming complex because the environment and societies are constantly changing. Meteorological services have used advanced technologies in early warning systems to alert people about extreme weather conditions. It should, however, be noted that before these technological advancements existed, indigenous communities around the world had warning systems for similar events and these early warning systems persist but are undocumented and threatened with extinction (Glantz 2009; Makwara 2013). The important aspect of any early warning system is that governments, the media and the affected people are warned to take action and must be receptive to the warning. The effectiveness of an early warning system, therefore, depends on how the users of the system perceive its strengths and weaknesses as this will determine whether the warning is accepted or not (Glantz 2009). Other factors, such as indigenous knowledge systems, may negatively impact the acceptance of early warning systems, as was documented in a study by the United Nations Environment Programme on indigenous knowledge in disaster management in Africa. The study showed that some communities in South Africa believe that hydrological hazards are released by specific deities in response to human misbehaviour (UNEP 2008). These attitudes are common in most indigenous communities and may have greater impact on community-based early warning systems that are expected to be participatory than on other types of early warning systems. However, all types of early warning systems have to deal with this challenge. An early warning system developed with the participation of communities is likely to be effective because the communities will identify with it and fully support its implementation. It should, however, be noted that not everyone in the community will necessarily have the same level of understanding and appreciation of an early warning system. Those involved in the formulation and organisation of the system may have a relatively better understanding of the system and hence participate more. CARE Philippines worked with local communities to create an early warning system for the A1 virus. Based on experiences with the process, they concluded that community members are their own subject matter experts and hence using village wisdom to create and implement early warning systems is the most effective way to create a successful system (CARE Philippines 2006). The early warning systems developed by meteorological services are usually global or national in nature. This is due to the complexity of the technology used and some nations rely on global warnings because of the non-availability of the equipment and capacity to utilise the equipment. The United Nations University has compiled a selection of climate, water and weather related to early warning systems. These early warning systems are mainly national and global. The hazards whose early warning systems are discussed in their book include hurricanes, cyclones, severe winter storms, heat waves, tornadoes, El Nino, severe weather air pollution, floods, droughts, famine, tsunamis, earthquakes, volcanoes and vector-borne diseases. They looked at case studies of early warning systems for each of these hazards. Their focus was the actual levels of warnings or the terms that forecasters used to inform the government and the public about the level of threat from a particular hazard. The vector-borne diseases documented include malaria and avian influenza. The early warning systems for avian influenza reviewed by the United Nations University are global and national. The shortcomings of such early warning systems are that the people at risk may not be able to receive and understand the hazardous phenomenon and the warning about it. Some of the global and national warning systems are provided in foreign languages or scientific terminology that may not be understood by the people at risk. Furthermore, the implementation of global early warning systems requires political will. If the government is not convinced about a global early warning system, it is unlikely that the warning will reach the people at risk. The MEWS discussed by the United Nations University is at national level with the collaboration between the Ministry of Health in Ethiopia and the National Meteorological Agency of Ethiopia. Despite the long-standing and well-known relationship between malaria and climatic variables, climate information had never been used to forecast malaria epidemics in Ethiopia. The communication in the system is such that when epidemics are foreseen, local authorities and nongovernmental organisations (NGOs) advise communities to implement preventive measures. We are of the opinion that, given the chance, communities can initiate warnings and not passively wait for information to come to them from higher levels. Even in the other early warning systems describe above, the potential in local communities is not tapped.

Other early warning systems that are documented include the pastoral early warning systems in the Greater Horn of Africa. According to a workshop held in 2001 on early warning systems, most of them have been designed to warn of drought-related crises even though the pastoralists are also exposed to other hazards. The early warning systems are largely funded by international donors and there is a perception that they service the information needs of these international agencies. This has serious implications on how sustainable the systems can be. When the organisations leave the respective countries, the systems are likely to collapse. It is important to note that early warning systems should be balanced in their approach. They should address the needs of the local communities, district and national governments as well as the international donors. Another lesson from the pastoralist early warning systems is that they should start at local level to the district and national level up to the international level. The movement upwards is determined by the exhaustion of the coping capacity of the preceding level.

Radice and Tekle (2011) have made the reasonable assertion that most of the global and national early warning systems focus on government-based warning information that is aimed at helping governments and donors and not the people affected by the hazard. The affected people, therefore, continue without adaptation strategies and coping mechanisms, and remain vulnerable despite repeated exposures to the same hazards.

#### Community-based early warning systems

Contrary to global and national early warning systems, community-based early warning systems empower communities to prepare for and confront hazards. According to the Pastoral Early Warning System document of 2006, communities are underinformed about the operation of early warning systems and underrepresented in the development of such systems. This results in early warning information that is disseminated but fails to trigger a response due to lack of community awareness and participation in response operations. In order to curb the above challenges, there is a need to develop community-based early warning systems. Community-based early warning systems are not synonymous with traditional early warning systems. We believe that traditional early warning systems or indigenous early warning systems fall under community-based systems because they are formulated and implemented by communities. The community-based early warning systems that have been identified from literature include: pastoral community-based early warning systems, flood and landslides warning systems, multi-hazard early warning systems, avian influenza warning systems and early warning system and mapping for an urban barangay. The majority of the community-based early warning systems documented in literature are for floods and landslides (Gautam & Phaiju 2013; Lassa & Sagala 2013; Phaiju & Bhandari 2012; Vanloon 2010).

From the analysis of the documented community-based early warning systems, it is evident that they were initiated by outsiders who took into consideration existing traditional knowledge. Almost all of the community-based early warning systems have been initiated or advocated for by NGOs, such as CARE Philippines, Save the Children UK Ethiopia, Practical Action, Christian Aid Social Action Centre, World Vision, USAID et cetera. The existence of national and global warning systems has reduced the communities to ‘at-risk’ communities without considering their capabilities in terms of early warning. According to Save the Children Ethiopia (2011), the main objective of community-based early warning systems is to transform at-risk communities into prepared disaster-resilient communities. The community-based early warning systems usually complement the national early warning systems rather than replacing them. This is because communities may not have the resources to respond to all types of disasters at the local level but can inform the national early warning system. Community-based early warning systems differ in terms of methodology, monitored indicators, complexities, timeliness and usability. This is usually determined by the implementing agencies, the disasters addressed and the affluence and setting of the community.

Mercy Corps and Practical Action (2010) described a community-based early warning system as that which is developed, managed and maintained by the community. The role of supporting organisations is to facilitate community participation. The early warning system is thus owned by the community. Mercy Corps and Practical Action (2010) further provided a more comprehensive outline of the features of a community-based early warning system. They argue that a community-based early warning system should involve all community members, especially the vulnerable groups at all stages of the CBEWS from designing to operating the systems, receiving the warning messages and responding to the warning. The measures taken should be based on the needs of everyone in the community, including the most vulnerable segments of the community so that the process and system is owned by the community members. Community-based early warning systems measures enhance the community members’ capacity to address their situation and participate in the decision-making process of EWS in a meaningful way.

The IFRC (2013) have done a comparative analysis between national early warning systems (NEWS) and community early warning systems (CEWS) in terms of their characteristics as shown in Table 1.

**TABLE 1:** Main characteristics of national early warning systems and community early warning systems.

Characteristics	National EWS>	Community EWS
Design	Deliberate, based on legal mandate by government or other agencies	Flexible design on need and adapted by trial and error
Human Resources	Technicians Specialists	Ad hoc volunteers to individuals appointed by local leaders
Characteristics	Formal staged warning Legislation Policies	Ad hoc and staged warning
Documented	Standard operating procedures MOUs Diagrammatic representations of information flow, etc.	Informal and rarely documented
Technology	High tech Access to telephones VHF HF radios	Telephone to traditional or no technology used
Trigger	Indicators, prediction, technology	Personal local detection of a hazard or receipt of a warning from outside the community
Warning process	Cascading or fanned in a systematic manner	Ad hoc, but may be naturally well organised and cascading/fanned
Messages	Impersonal	Personal
Timing	Not always the first to be received by community Produced to share with official systems at all levels	Rapid (when message created at community level) or co when there are good linkages between all levels
Primary needs targeted	Reduce economic and other losses	Safety, reduce stress, emotional support
Evaluation criteria	Hazard details Lead-time provided Proportion of false warnings	Timeliness of receipt of warning, actionable message in warning

*Source*: International Federation of Red Cross and Red Crescent Societies, 2013, ‘Community early warning systems: guiding principles’ Community Preparedness and Risk reduction Department Geneva, viewed 28 February 2015, from http://www.ifrc.org/pageFiles/103323/1227800-IFRC-CEW-Guiding-Principles-EN.pdf EWS, early warning systems; MOUs., Memoranda of understanding; VHF, Very High Frequency; HF; High Frequency.

The Red Cross and Red Crescent describe traditional early warning systems or indigenous early warning systems rather than community-based early warning system. As mentioned before, we believe that traditional early warning systems are under community-based systems. Our opinion is that for a system to be community-based, it has to be initiated at community level and operated by the communities themselves. Being community-based, does not necessarily mean that it has to be informal. A community-based early warning system can be formal and well documented. It can also use technology, depending on the affluence of the community and the setting in which the system is operated. Gonzales *et al*. (2013) designed a community-based early warning system in Leveriza Manila in which the communities had access to the use of the technologies like cellular phones, computers and Internet connection. The system had the following features: utilisation of short messaging services, Google Maps social networking sites, Wiki pages and the generation of reports. This is an example of the use of technology in a community-based early warning system. Community-based early warning systems can also be designed by NGOs working with the communities as in the flood warning systems in the Philippines. (Perez *et al*. 2007). Community-based early warning systems may or may not have characteristics similar to a national early warning system. The difference is mainly at the level of operation. We agree with the characteristics of CEWS described in Table 1 if the meaning of CBEWS is the same as traditional or indigenous early warning systems. Such CBEWS are therefore generally informal and do not use complex technology.

Radice and Tekle (2011), made the important observation that community-based early warning systems should be active in normal times not only during emergencies. This means communities should be able to see the benefit of the systems and be able to collect, analyse and disseminate the information themselves. We believe that early warning systems that are active during normal times are the ideal systems because they can predict imminent danger rather than being initiated when the disaster has already occurred. That is more surveillance rather than early warning.

#### Methodologies or characteristics of selected community based early warning systems

Some documented community-based early warning systems were analysed to determine the implementing agencies, the hazards addressed, the methodologies used in their development or the characteristics of the systems and finally their strengths and weaknesses. Table 2 summarises the analysis. As already mentioned, most of the community-based early warning systems were initiated and implemented by NGOs. The majority of the documented community-based early warning systems are for natural disasters, flood warning systems most of all. Mercy Corps and Practical Action (2010) and also CARE Philippines (2006) provide step-by-step methodologies for the development of community-based early warning systems. A closer look at the two systems by CARE Philippines and Mercy Corps and Practical Action shows that one was derived from the other. It is most likely that the methodology for the avian influenza early warning system was derived from that of the flood early warning system. The rest of the systems show a description of the characteristics of the community-based early warning systems from which methodologies for their development can be drawn.

**TABLE 2:** Methodologies or characteristics of selected community based early warning systems.

Implementing agency	Hazard	Methodology or characteristics	Strengths	Weaknesses
Save the Children Ethiopia	Drought (designed for pastoralists)	Taping of existing organisational structures and mechanisms within communities Identification of local actors, Participatory Analysis (hazard mapping, vulnerability assessment etc.) Community mobilisation and volunteerism, implementation of DRR Measures Development of food security Monitoring systems and communication dissemination plans	Low cost Low tech Relevant to the communities Community participation Sustainable uses existing structures	Traditional indicators not recognised
Mercy Corps and Practical Action	Floods	Step 0 preparatory step Step I participatory situational Analysis and formulation of the early warning management committee Step II Development of Observation and monitoring system with the active participation of the community Step III development of a communication and dissemination plan Step IV preparation for response Step V follow up, review and Sustainability	Comprehensive and easy to follow Participatory community centred Outsiders only facilitators Promotes sustainability of the system Takes into Account the existing indigenous early warning systems	Biased towards flood disasters
CARE Philippines	Birds flu	Step I avian influenza preparedness orientation Step II formation of five preparedness teams; (surveillance, information and education team, Communications, survey quarantine team and rapid action team); the composition tasks and function of each team are clarified Step III follow-up sessions on Early Warning System are conducted with the Preparedness and Response. teams (review signs and symptoms of avian influenza in animals and in humans to ensure early detection, introduce and fill up monitoring and incident reporting form, clarify reporting flow, rehearse key preparedness messages for public information, practice dissemination of information, updates, and corresponding emergency measures that the community will take if there are cases of avian influenza Step IV Involves agreement on the mechanics of setting up and maintaining the Early Warning System using the 4 Ws (what, where, when and who) Step V Public information activities Step VI review of the system after 6 months and incorporation of any changes that are necessary	Comprehensive, easy to follow, has a communication and education component Capacity building on the communities Gives room for clarifications and rehearsals before implementation of the system Utilisation of existing structures	Disease specific
Information Technology Department De La Salle University, Manila	Natural Disasters in general	Rapid Application Development (RAD an approach in systems development that assures better and more inexpensive systems and more rapid operation by having systems developers and end users work together jointly in real time to develop systems (Hofer, George & Valacich 2002)	User involvement, Interaction with users during development, caters for Different methods of information dissemination not only disasters, system consolidates information on disaster histories Provide accurate reports	Limited to communities with access to Internet and mobile phones communicates only to registered users Network problems SMS sending delayed due to network traffic Inherent problems with twitter and Facebook may affect the system
Philippine Atmospheric, Geographical and astronomical Services Administration	Floods	Methodology not outlined but involves: Decision by communities to have a CBEW; consultation of community members and selection of volunteers; establishment of rainfall and water level stations; establishment of threshold values for rainfall observation; observations by volunteers; drills	Simple, inexpensive Incorporates indigenous knowledge of communities Promotes ownership thus sustainability Political commitment	Methodology not clearly outlined, not easy to adapt Very specific to floods
Department of Hydrology and meteorology, Nepal, Practical Action, local Government and nongovernmental organisations	Floods	Area survey, flood hazard Mapping and assessment of warning level and danger level; installation of monitoring instruments; setting up of an operation centre; development of a communication and warning dissemination system; training and capacity building cooperation with NGOs working in the area	Utilises existing communication channels Collaboration with other stakeholders working in the area Participation of vulnerable groups. Role of community was clearly outlined	Specific to floods
Social Action	Floods	Setting up two way radio communication system, establishment of flood watch points, installation of manual rain gauges, collaboration with existing stakeholders, training of the disaster coordinating council, establishment of linkages with scientific agencies, communication of climate forecasts to communities	Collaboration with other stake holders capacity building utilisation of community volunteers	Does not take into consideration indigenous methods, top-down approach, initiated by NGOs and brought to the people, limited community participation
Centre Prelature of Infanta World Vision	Landslides and mudslides multi-hazard	Training, hazard identification, hazard analysis, vulnerability analysis, early warning/ community surveillance mitigation plan and implementation, preparedness and response, monitoring and evaluation, community awareness on available resources, communication system	Community participation from inception, takes into account indigenous knowledge, sustainability is addressed Identification of existing resources to address hazards bottom-up and top-down communication	Addressing many hazards in one system, though desirable may compromise focus on some of the hazards Methodology not exclusive for early warning, general community awareness activities not clear
Practical Action	Floods	Community awareness and Sensitisation, task groups and volunteers, flood monitoring both automatic and manual at different strategic locations, dissemination of information at all levels; institutions, individuals and networks, Response to information, preparedness for response village and district level, rescue and evacuation trainings, mock drills	Community participation, capacity building, sustainability utilises existing structures, uses available technology e.g. radio, telephone	Does not take into account existing systems and does not consider indigenous knowledge
Oxfam	Drought	Interactive participation, needs assessment, community action plans, formation/strengthening of community groups, awareness creation	Community participation utilisation of local resources community empowerment strengthening of existing structures external support seen as complementary or gap filling	Does not take into account existing early warning systems
Christian Aid	Floods	Use of civil protection committees at village level, communication using megaphone and posters at strategic points, installation of weather measuring equipment, community awareness	Utilisation of existing structures Community awareness Community participation in development and utilisation of the system	Not clear on utilisation of indigenous systems and community participation Methodology not clearly outlined, outline of implementation rather than methodology.
USAID	Floods	Existing available warning Information collected together with planned information in future, early warning packaging, early warning needs assessment, consultation with stakeholders sensitisation of communities	Both top-down and bottom-up communication Takes into account existing structures	Sustainability not clear
Centre for International Studies and Cooperation, Vietnam	Floods	Empowerment approach, community participation, learning by doing, training and mentoring , cooperation and shared responsibility, participation of vulnerable groups, long-term relationship building, simple tech solutions, monitoring and evaluation	Sustainability, cultural relevance, collaboration between nongovernmental organisations Local government and villagers	Biased towards floods

DRR, Disater Risk Reduction.

The major strengths of all the systems analysed are that they are low cost, relevant to the communities and promote sustainability. Identified weaknesses are that some of them are not easy to adapt for disasters other than those for which they were originally formulated. Some of the systems, even though they are community-based early warning systems, do not take into account the existing traditional indicators that the communities may have. Disregarding the knowledge that exists within the communities may have implications for sustainability. The ideal would be to integrate new knowledge and existing knowledge. Technology-based systems pose problems in that many people may not have access to the technology, such that the utilisation of such systems is limited to users who do have access.

The methodology for the avian influenza early warning system by CARE Philippines and the flood early warning system by Practical Action and Mercy Corps can be adapted for the development of a community-based MEWS. The details for each system are shown in Table 2.

## Discussion and conclusion

The community-based early warning systems that are documented are mainly for natural disasters. In the reviewed literature, no community-based early warning system has been suggested for malaria. According to Chaiken (2008), shocks in communities are usually associated with severe sudden and catastrophic events such as earthquakes, hurricanes or large-scale floods. However, she argues that the most difficult shocks to deal with are those that are chronic and slow to emerge, for example, endemic malaria.

There is generally no documentation of the initiatives in the development of community-based early warning systems for human diseases, except the one by CARE for avian influenza, (CARE Philippines 2006). Diseases like malaria have existed for a long time and are influenced by weather parameters that communities can observe and predict. The lack of community-based MEWS may be attributed to the perception that malaria is not a natural disaster similar to floods and earthquakes. The capacity of the local community to predict the disease has been undermined. The top-down approach in the malaria control programmes may also have contributed to the lack of community participation in the development of MEWS. The prediction of malaria has been viewed as complex and thus leaving no room for the simple methods used by communities to predict diseases.

The successes in community-based early warning systems for weather-based disasters indicate a potential for the development of early warning systems for diseases that are influenced by weather. The establishment and implementation of the community-based early warning systems differ from setting to setting, but have a common conceptual framework that includes the involvement of existing organisational structures and mechanisms within communities (the identification of local actors); participatory analysis by means of hazard mapping, vulnerability and capacity assessments, community DRR planning, etc.; community mobilisation and volunteerism, implementation of DRR measures (both non-structural and structural) and the development of monitoring systems, and communication and dissemination plans.

The United Nations International Strategy for Disaster Reduction acknowledges that simple technologies, such as rainfall and river gauges, combined with equally simple rules of thumb can often enable communities to monitor threats and provide effective warnings. This shows the possibility of the utilisation of indigenous knowledge systems in the development of early warning systems especially in settings where the conventional or formal early warning systems are not reaching the affected populations. Diseases like malaria that are affected by weather conditions can give communities ample time to prepare for the hazards after prediction of the seasons.

The case studies presented show that NGOs have taken the lead in development of community-based early warning systems. The IFRC has developed guiding principles for CEWS. The IFRC simplified and slightly adapted the four core interlocking components of an early warning system published by the UNISDR. In their guidelines, they provide examples of agencies that engage in global or regional early warning monitoring. The hazards that they documented include severe weather, flooding and landslides, drought, wild land fire, earthquakes, volcanoes, tsunamis, epidemics and conflict. They warn that setting up an early warning system at any level without clear links to other disaster reduction and management efforts and entities will result in inefficient, unsustainable products and less effective impact. The setting up of CBEWS requires a considerable investment of time and resources and should not be undertaken without considering alternatives. They encourage synergy across levels, i.e. community, national, regional and/or global. The FIRC has designed guiding principles per each early warning component for community-level practice. They did not prescribe a method for the establishment of the system but rather provided principles on which CBEWS should be based. The guidelines of the IFRC recognise risk knowledge as the foundation of early warning. They emphasise that implementation agencies should accept that community priorities may not be their priorities. Early warning systems should have a monitoring mechanism that evolves as the hazards evolve. Communities need to drive their own early warning systems since passive receivers will not save lives and they can be motivated through public displays of monitoring activities. The guidelines emphasise the response to warnings and not disasters, and therefore the need to have embedded response options in annually updated contingency plans with links to funding. The response actions should be tested in order to achieve robust no-regrets response actions. In terms of warning communication, the guidelines advocate the clear delegation of responsibilities to alert or mediate the hazard. The utilisation of sophisticated warning devises should be avoided. The IFRC puts across an argument on the terminology in early warning systems with the suggestion that the term ‘community’-based early warning systems’ does not really imply community participation but may mean a system that is based at community level but implemented by other agencies. The ideal term would be ‘CEWS’. This term describes the systems more appropriately in that it gives the impression that the systems belong to the communities. The United Nations Development Programme (UNDP) and the European Commission Humanitarian Office, through their disaster preparedness programme, has compiled a review and analysis for community-based practices for reducing the risk of disasters from natural hazards affecting South East Africa and South Western Indian Ocean. The review and analysis focuses on floods caused by swelling rivers; cyclones storm surges, earthquakes, fire and other such events.

The community-based early warning systems methodologies reviewed are all based on the same principles that encompass that the methods include simplicity, be community driven, include vulnerable groups, use local capacities for warning and response and advocate ownership of the systems by the communities. These principles are actually a simplified form of the UNISDR elements of early warning systems. It is clear that there is no one method of designing early warning systems. The principles are mainly guidelines that communities and organisations can use to develop their own systems. The methodology adopted mainly depends on the setting and also the type of disaster that is addressed.

**Conclusion**

The review has revealed that the concept of early warning encompasses the issuance of the warning, the response and feedback. The definitions of early warning systems found in literature are mainly centred on the UNISDR 2006 definition. Community-based early warning systems have been developed mainly for natural disasters. There are no community-based early warning systems for malaria that have been documented in literature. The same principles used for the development of community-based early warning systems for natural disasters may be utilised in the development of community-based early warning systems for communicable diseases. This was demonstrated by CARE Philippines (2006). There is, therefore, scope for further work to be done in the development of community-based early warning systems for malaria.
